# Examining Perceptions about Mandatory Influenza Vaccination of Healthcare Workers through Online Comments on News Stories

**DOI:** 10.1371/journal.pone.0129993

**Published:** 2015-06-18

**Authors:** Yang Lei, Jennifer A. Pereira, Susan Quach, Julie A. Bettinger, Jeffrey C. Kwong, Kimberly Corace, Gary Garber, Yael Feinberg, Maryse Guay, Public Health Agency of Canada/Canadian Institutes of Health Research Influenza Research Network (PCIRN) Program Delivery and Evaluation Group

**Affiliations:** 1 Public Health Ontario, Toronto, ON, Canada; 2 University of Toronto Faculty of Medicine, Toronto, ON, Canada; 3 Vaccine Evaluation Center, Vancouver, BC, Canada; 4 BC Children’s Hospital, Vancouver, BC, Canada; 5 University of British Columbia, Vancouver, BC, Canada; 6 Dalla Lana School of Public Health, University of Toronto, Toronto, ON, Canada; 7 Institute for Clinical Evaluative Sciences, Toronto, ON, Canada; 8 Department of Family and Community Medicine, University of Toronto, Toronto, ON, Canada; 9 University Health Network, Toronto, ON, Canada; 10 The Ottawa Hospital, Ottawa, ON, Canada; 11 University of Ottawa, Ottawa, ON, Canada; 12 University of Ottawa Institute of Mental Health Research, Ottawa, ON, Canada; 13 Département des sciences de la santé communautaire, Université de Sherbrooke, Longueuil, QC, Canada; 14 Institut national de santé publique du Québec, Longueuil, QC, Canada; 15 Centre de recherche de l’Hôpital Charles LeMoyne, Longueuil, QC, Canada; 16 Agence de la santé et des services sociaux de la Montérégie, Longueuil, QC, Canada; University of Waterloo, CANADA

## Abstract

**Background:**

The aim of this study was to understand online public perceptions of the debate surrounding the choice of annual influenza vaccinations or wearing masks as a condition of employment for healthcare workers, such as the one enacted in British Columbia in August 2012.

**Methods:**

Four national and 82 local (British Columbia) Canadian online news sites were searched for articles posted between August 2012 and May 2013 containing the words “healthcare workers” and “mandatory influenza vaccinations/immunizations” or “mandatory flu shots and healthcare workers.” We included articles from sources that predominantly concerned our topic of interest and that generated reader comments. Two researchers coded the unedited comments using thematic analysis, categorizing codes to allow themes to emerge. In addition to themes, the comments were categorized by: 1) sentiment towards influenza vaccines; 2) support for mandatory vaccination policies; 3) citing of reference materials or statistics; 4) self-identified health-care worker status; and 5) sharing of a personal story.

**Results:**

1163 comments made by 648 commenters responding to 36 articles were analyzed. Popular themes included concerns about freedom of choice, vaccine effectiveness, patient safety, and distrust in government, public health, and the pharmaceutical industry. Almost half (48%) of commenters expressed a negative sentiment toward the influenza vaccine, 28% were positive, 20% were neutral, and 4% expressed mixed sentiment. Of those who commented on the policy, 75% did not support the condition to work policy, while 25% were in favour. Of the commenters, 11% self-identified as healthcare workers, 13% shared personal stories, and 18% cited a reference or statistic.

**Interpretation:**

The perception of the influenza vaccine in the comment sections of online news sites is fairly poor. Public health agencies should consider including online forums, comment sections, and social media sites as part of their communication channels to correct misinformation regarding the benefits of HCW influenza immunization and the effectiveness of the vaccine.

## Background

Seasonal influenza carries a high burden of disease, especially in persons aged ≥65 years or <2 years [1–3]. Vaccinating health care workers (HCWs) against influenza is an effective strategy to prevent transmission in healthcare settings [4]. Yet despite the widespread availability of the vaccine and strong recommendations for HCW influenza immunization, coverage in many Canadian healthcare organizations is low (40–60%) [5–7].

Some healthcare institutions in the United States have introduced policies making influenza vaccination a condition of work for HCWs, leading to vaccine coverage levels exceeding 90% [8–12]. In 2000, the Ontario Ministry of Health and Long-Term Care attempted to legislate mandatory influenza vaccination for paramedics, a policy that was repealed after HCWs challenged it for violating personal autonomy. No other Canadian province tried to implement mandatory vaccination until 2012, when the British Columbia (BC) Ministry of Health announced a policy of either influenza vaccination or wearing a mask during influenza season as a condition of service for HCWs [13]. Elsewhere in Canada, Horizon Health Network, one of the two health authorities in New Brunswick, implemented a similar policy in 2012, as did 13 Ontario hospitals in 2013 and 12 Toronto-area academic hospitals in 2014 [14], and the province of Saskatchewan in September 2014[15]. The policies in Ontario, New Brunswick, and Saskatchewan have not been legally challenged and are still in place. However, the province-wide BC condition of service policy was controversial among HCWs and resulted in a legal challenge by the nursing union. The policy was upheld in arbitration in October 2013[16].

Given the media attention around the BC policy (S1 Table), it is important to understand the perceptions of the general public towards influenza vaccination as a condition of service for HCWs. This is because online comments may influence the opinions of other content consumers—including HCWs—through the effects of exemplification [17–20], and may subsequently erode confidence in vaccines in general. We chose to focus on the comment sections of online newspapers since nearly 75% of Canadians read a newspaper each week, of which 42% read online content [21]. The advantages of analyzing perception data derived from social media are that they are readily available and can be drawn from a large geographic area [22]. Readers posting on major news sites may be more honest about their opinions since a certain degree of anonymity can be maintained. In addition, compared to other forms of social media such as Twitter, users can post more detailed opinions without restriction on the number of characters. The disadvantages include the fact that the population of online commenter do not necessarily reflect the general population, or HCWs, and that more negative voices tend to be more vocal online [23]. To understand Canadians’ perceptions of the seasonal influenza vaccination as a condition of service for HCWs, we evaluated readers’ responses to online newspaper articles after the BC policy was announced but before it was upheld in arbitration [16].

## Methods

### Data sources

**Fig 1:**
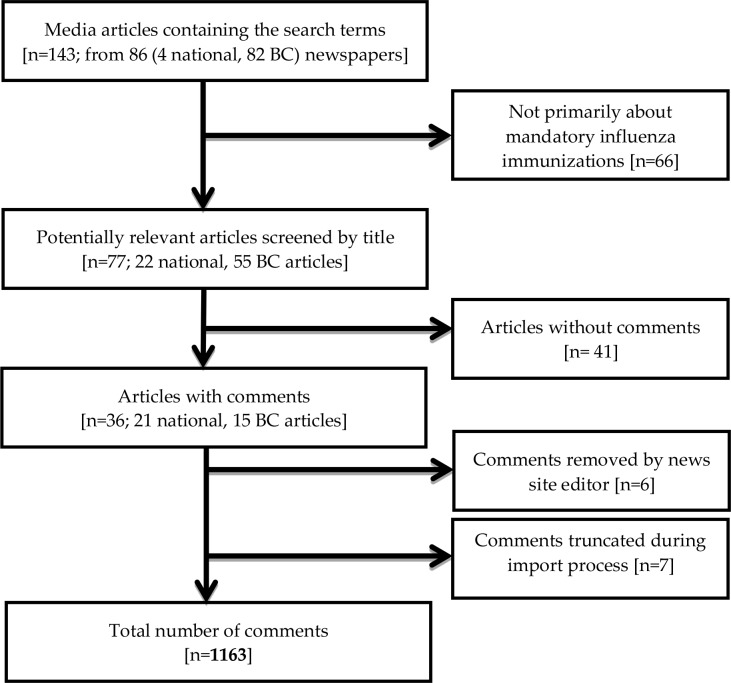
Search strategy and inclusion criteria. An initial search returned 143 articles from which we filtered down to 36 articles containing 1163 comments.

We searched four national and 82 local (BC) Canadian online English language news sites for news articles about mandatory influenza vaccination policies for HCWs (Fig 1). We focused on BC, given that the condition of service policy was introduced province-wide for the 2012–13 influenza season, and therefore the majority of press coverage would be from that province. We used an online newspaper database to identify BC news sites [24].

We considered English news articles posted between when the policy was announced by the Provincial Health Officer, on August 23, 2012, and the last day before our analysis began, on May 31, 2013, inclusive. We only considered English language articles since the Francophone community in BC is very small and the topic was not covered by newspapers in Quebec. Articles were included if they contained the words “*healthcare worker*” and “*mandatory influenza vaccinations/immunizations*” or “*mandatory flu shots and healthcare workers*.” Each article was reviewed to ensure that they predominantly pertained to the topic of mandatory influenza vaccinations for HCWs and had at least one comment.

### Analysis

#### Descriptive analysis

We summarized descriptive statistics for sentiment toward both seasonal influenza vaccines and the BC condition of work policy, and number of comments per individual. Individuals were defined as having a unique profile name associated with their comment. Some news sites allowed readers to enter comments under a “Guest” profile. For these comments, we counted each “Guest” as a unique individual. For each comment, we recorded whether it included an influenza vaccine-related statistic or a link to a website, whether it contained a personal story, and whether the individual self-identified as a HCW. We combined multiple comments from the same username on the same article as one user. In reading the first 50 comments, we identified three main patterns, and began to categorize each user into three groups: 1) those who do not believe in the effectiveness of seasonal influenza vaccines, and do not support vaccination as a condition of service for HCWs; 2) those who do believe in the effectiveness of seasonal influenza vaccines, and do support vaccination as a condition of service for HCWs; and 3) those who do believe in the effectiveness of seasonal influenza vaccines, but do not support vaccination as a condition of service for HCWs.

#### Qualitative analysis

Using the process of thematic analysis [25], two researchers (S.Q. and Y.L.) co-coded 20% of the unedited comments independently, generating a coding dictionary that was based on the first 50 comments, and adding codes as required. After conferring and reaching consensus on the finalized coding list, S.Q. coded 30% and Y.L. coded 70% of the remaining comments. Next, the codes were organized into themes and the research team reviewed the results together to ensure that both clinical and methodological perspectives were brought to the analysis. Each comment could contain multiple themes. The length of the comment was not considered. All analyses were conducted using QSR NVivo 10.

## Results

Our online searches of media sites identified 36 articles that met our inclusion criteria, with 21 national articles, and 15 articles from BC newspapers (Fig 1). The majority of the articles were news articles (n = 32), with the remaining four being opinion pieces. Of these four, two supported the vaccination policy, one was against, and one was neutral.

From these 36 articles (S1 Table), we analyzed 1163 comments from 648 individuals (1.8 comments/person). The majority (900/1163) of comments came from national news sites. Most newspapers moderated the comment section, but only 20/1163 comments were removed by moderators pre-analysis. Of the 648 individuals, 182 (28%) expressed positive perceptions of influenza vaccines (1.7 comments/individual), 313 (48%) expressed negative perceptions (1.8 comments/individual), 25 (4%) had mixed feelings (1.3 comments/individual), and 128 (20%) were neutral (2.0 comments/individual).

Eighty-three individuals (13%) cited personal stories in their comments, 70 (11%) self-identified as healthcare workers, 144 (22%) supported a condition of service influenza vaccine policy like the one proposed in BC, 69 (11%) were neutral to such a policy, and 435 (67%) were against such a policy (Table 1). A total of 163 (25%) users provided links to information sources or statistics in their comments. The most commonly cited sources are noted in the S1 Fig. The proportion of comments and individuals expressing the different sentiments were not substantially different between the entire dataset and the subgroups examined, with the exception of users who provided a link or statistic in their comment. These users had a higher rate of negative sentiment toward the influenza vaccine than the other groups (68% vs 45%-51%) (S2 Table). Of the newspapers analyzed, only the Globe and Mail and National Post publish their online readership demographic data. Analysis of the themes by individual newspapers (Globe and Mail, National Post, CBC, CTV, BC local papers) did not show them to be substantially different from the themes that emerged from the data as a whole (Table 1).

**Table 1:** Comparison of sentiment towards influenza vaccine and indicators of interest between commenters to articles in B.C. papers and national papers.

		Number of individuals (%) (Overall)	Number of individuals (%) (B.C.)	Number of individuals (%) (Canada)
	N	648	92	556
**Sentiment toward influenza vaccine**	**Positive**	182 (28%)	19 (29%)	163 (29%)
	**Negative**	313 (48%)	41 (45%)	272 (49%)
	**Neutral**	128 (20%)	27 (29%)	101 (18%)
	**Mixed**	25 (4%)	5 (5%)	20 (4%)
**Indicator of interest**	**HCW**	70 (11%)	11 (12%)	59 (11%)
	**Personal Story**	83 (13%)	10 (11%)	73 (13%)
	**Link or Statistic**	114 (18%)	22 (24%)	92 (17%)
	**Support for B.C. Policy**	144 (22%)	16 (17%)	128 (23%)

### Perceptions of Influenza Vaccine and Condition of Service for HCW

Most individuals fell into the following three categories: 1) those who did not believe in the effectiveness of seasonal influenza vaccines and did not support vaccination as a condition of service for HCWs (48%); 2) those who did believe in the effectiveness of seasonal influenza vaccines and did support vaccination as a condition of service for HCWs (22%); and 3) those who did believe in the effectiveness of seasonal influenza vaccines, but did not support vaccination as a condition of service for HCWs (6%). The remaining 24% of commenters did not express an opinion on the effectiveness of the seasonal influenza vaccine.

**Fig 2:**
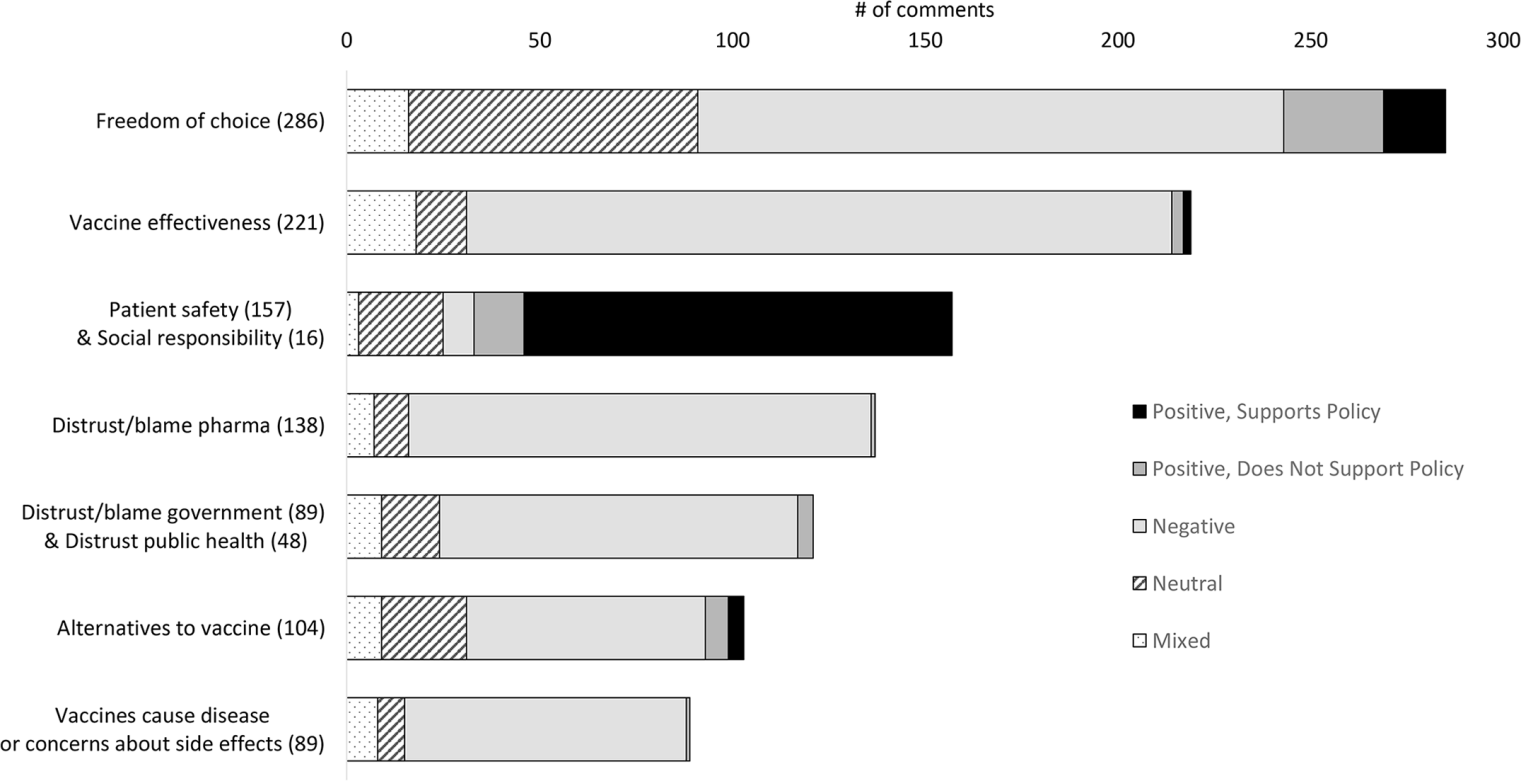
Seven most frequent themes and their distribution of sentiment. Freedom of choice, vaccine effectiveness, patient safety, distrust of the pharmaceutical industry, distrust of the government or public health, alternatives to vaccine, and concerns about side effects are the seven most frequently-appearing themes in our analysis.

The distribution of commenter sentiment of the most frequent themes is shown in Fig 2.

#### Freedom of choice

The most common theme (286 comments) was *freedom of choice*. Many commenters incorrectly perceived the BC policy as making influenza vaccination mandatory for HCWs, viewing it as a state intrusion into private lives and an erosion of civil liberties. Others agreed with the intent of the policy but thought the (incorrectly perceived) lack of choice would be seen as excessive, while some felt it was justified on the grounds of patient safety. Of commenters who understood that the policy allowed for mask-wearing as an alternative to the vaccine, some felt this to be fair, while others argued that the option of wearing a mask during influenza season was designed to identify and intimidate HCWs who did not receive the vaccine. Commenters also questioned the effectiveness of masks at preventing influenza transmission as well as the ability of HCWs to tolerate wearing masks for the duration of their shifts.

“I agree it is a good idea for healthcare workers to get vaccinated. However people have fought a long time for the right to decide what happens to their own body, this is not your [employer’s] choice. Where does it stop if we give our employer these [rights] to our bodies? Women must have tubes tied after 1 child otherwise they are away from work too long if they have more children? Or maybe hospital workers must have [tattoos] to explain their entire medical history to comfort patients. Same argument. Your body your choice.” “Dr. Grand”, in response to http://www.cbc.ca/news/canada/thunder-bay/story/2013/01/30/tby-hospital-flu-shot-debate-thunderbay.html.

#### Vaccine effectiveness

The second most common theme (221 comments) was *vaccine effectiveness* **,** with many commenters questioning the effectiveness of influenza vaccines. Some commenters rejected the concept of immunization in general, while many supported vaccines for other infectious diseases such as polio, measles, mumps, rubella, and smallpox. Those who discussed vaccine effectiveness frequently referred to sources such as the Cochrane Collaboration and the University of Minnesota Centre for Infectious Disease Research and Policy (CIDRAP). A smaller group of commenters believed that seasonal influenza vaccines are effective in preventing influenza and its spread. Commenters questioned the credibility of sources that others on the online boards had used to support their claims. Allegations of bias in sources were frequent, and some commenters stated that more information and research on influenza vaccines was needed.

“Get the shot, wear a mask, or go home. You are working with sick people, many with compromised immune systems. The shot takes only a moment, and you're done. If you have a problem with needles, perhaps you shouldn't be in mainstream public healthcare. It's not draconian, only a responsible reaction on the part of the hospital administration.” “Dendrast”, in response to http://www.cbc.ca/news/canada/thunder-bay/story/2013/01/30/tby-hospital-flu-shot-debate-thunderbay.html.

“As an RN I am offended by comments that suggest refusing the shot translates to a crappy nurse. Until peer reviewed research confirms I am a risk to patients, I will NOT get the shot and I am happy to stay home without pay. What other profession demands the worker to knowingly chemically alter their body with potentially insidious risks? Anyone care to know how the "shot" is made? Based on predictions they throw a cocktail together. EVERY year they have missed the mark, yet they still bully workers into having it. For you know-it-all, believe everything you hear people, I remind you of one of thousands of medical blunders. the thalidomide tragedy—a "wonder drug" endorsed by physicians, encouraged by health authorities and permitted to kill and deform babies for 10 years before discontinued. The drug was developed and used in Nazi prison camps and fed to Canadians post war. Minus the prison camp—is there a difference in the mandatory imposition of these two drugs?” “RNnotPOW”, in response to http://news.nationalpost.com/2013/01/24/nurses-aide-sent-home-for-refusing-flu-shot-the-latest-healthcare-worker-suspended-in-push-to-make-immunization-mandatory/.

#### Patient safety and social responsibility

Many commenters (173 comments) advocated for mandatory influenza vaccination on the basis of *patient safety and social responsibility*. They argued that regardless of the benefit to the person receiving the vaccine, strengthening herd immunity against the influenza in hospitals and long-term care facilities indirectly protects patients with less robust immune systems. These commenters felt that there was a duty to reduce risk to vulnerable patients. Critics replied that HCWs were only a subset of those inside a hospital at any given time, and that herd immunity is impossible given the presence of new patients or visitors with influenza.

#### Distrust of authorities

Many commenters (270 comments) expressed a *distrust of authorities*, including government, public health, and pharmaceutical companies. Some commenters speculated on whether the government would receive kickbacks from the pharmaceutical companies, since the vaccine is required annually and enacting a mandatory policy would guarantee a certain number of orders each year. Others suspected the policy was put in place not to reduce risk to patients but to reduce sick time for HCWs, thereby saving money. Several commenters saw HCWs as role models and questioned their low rates of influenza vaccination. Finally, a small number of commenters distrusted the quality of watchdog activities and inspections undertaken by Health Canada to ensure vaccine safety and effectiveness. Commenters about the pharmaceutical companies’ role in the issue were universally negative, with sentiments ranging from resignation to outright accusations of deliberately endangering the public for profit. Some commenters believed that pharmaceutical companies’ refusal to offer compensation for adverse drug reactions in Canada was suspicious, given that compensation was offered in several other countries.

#### Alternatives to vaccination

Many commenters (104 comments) advocated non-vaccine related methods of reducing risk of influenza. These *alternatives to vaccination* included physical barriers to transmission (e.g., masks), improved hygiene (e.g., proper hand-washing by hospital staff), social distancing (e.g., staying home when sick), and constitutional fortification (e.g., better nutrition, vitamin C, regular exercise). Some commenters suggested these alternatives as a supplement to vaccination, while others denounced vaccines completely and advocated only these alternatives.

#### Vaccine safety

Several commenters (89 comments) had concerns about the *safety of vaccines*. Some felt all vaccines were unsafe, fearing the effects of vaccine ingredients such as thimerosal and formaldehyde. Many expressed concerns about immune reactions, from rashes and allergies to Guillain-Barré Syndrome, and some believed, or told personal stories of the influenza vaccine itself transmitting influenza. Several commenters cited an article by a BC researcher (Skowronski et al.) that suggested that receipt of the 2008–09 seasonal influenza vaccine made patients more vulnerable to the 2009 H1N1 pandemic influenza virus [26].

#### Influenza is a minor illness

A small number of commenters (< 30 comments) believed that *influenza was a minor illness*, and vaccination as a condition of service would result in over-vaccination and a dependence on drugs and vaccines. Most of these individuals agreed that the vaccine was necessary for specific vulnerable populations, such as patients with compromised immune systems.

Some commenters questioned the effectiveness of mandatory vaccination for just a segment of the population, given community transmission of influenza. They debated the value of vaccination as a condition of service for HCWs only given that HCWs are exposed to influenza in other non-healthcare settings.

## Discussion

The “influenza vaccine or mask” condition of service policy for HCWs implemented by the BC Ministry of Health in August 2012 generated reader comments to many Canadian online news articles. The main themes of these reader comments included: (i) freedom of choice, (ii) vaccine effectiveness, (iii) patient safety and social responsibility, (iv) distrust of government, public health, and pharmaceutical companies, (v) alternatives to vaccines, (vi) and concerns about vaccine safety. The majority of the commenters did not support the BC condition of service policy, and a plurality of commenters harboured a negative view of influenza vaccines.

In this study, approximately 48% of individuals did not believe in the effectiveness of influenza vaccination and did not support the condition of service policy, while only 22% of individuals both believed in the effectiveness of the vaccine and supported the condition of service policy. This suggests that the overall perception of the influenza vaccine and support for mandatory influenza immunization was poor among those readers who provided comments. This is not surprising given that many studies have also documented negative attitudes towards influenza vaccines among HCWs and the general population [27–29]. Commonly cited reasons for refusal of the vaccine include misconceptions regarding the effectiveness and safety of the vaccine, as well as a belief that the vaccine is unnecessary because influenza is not a serious illness [30–33].

While we anticipated that there would be strong negative sentiment against both influenza vaccines and the BC policy, we were surprised by just how vocal this group was. We were also surprised by the subset of commenters who had positive sentiment toward the influenza vaccine but did not support the BC policy. We did not see much discussion about influenza being a minor illness. This may be because the BC policy aroused stronger emotions about labour relations and thus shifted the discussion in that direction and away from influenza itself. Many of the negative comments we found appear to be rooted in misunderstanding the influenza vaccine and the condition of service policy. This is particularly important because comments have been shown to influence other readers’ perception of the issue, such that a glut of negative comments on an article may sway a previously neutral reader’s opinions [17–19]. Commonly sourced journal articles are often split in their recommendations about whether future efforts of public health should be focused on increasing influenza vaccine coverage or developing a more efficacious vaccine [34–36]. Some commenters have illogically but loudly used this as evidence that the influenza vaccine is not effective. Some research has also suggested that online forums are a popular outlet for negative sentiments to proliferate and that these stories are more frequently shared than positive ones in social media [37]. Real-time responses to negative comments could have a role in slowing the propagation of erroneous ideas about future public health announcements.

For many commenters, the belief that influenza vaccines are ineffective is linked to a distrust of the government, public health, and pharmaceutical companies. Many users believe, even if they have positive sentiments towards influenza vaccines and support the BC policy, that the government or public health officials have inappropriate ties with the pharmaceutical industry. A previous study that examined public perceptions of measles using a similar methodology also found a strong distrust with these bodies [38], suggesting that this is not solely an issue of influenza vaccines but a broader topic about vaccines and government-industry relations in general. Therefore, in the initial public health messaging of similar future policies, healthcare organizations should be clear about the relationship between government and the pharmaceutical industry to counter public mistrust. Studies of attitudes of both HCWs who worked at organizations with mandatory vaccination or alternative programs (such as mandatory vaccine or mask policies) and those who worked at organizations with voluntary programs reflect similar themes [39–41].

## Limitations

The major limitation of this study is that the online commenting population does not necessarily reflect the readership of each news source, nor of the general population. Individuals who are most likely to comment are those with particularly strong (and often negative) opinions [23]. Further, although we treated each unique username as a unique person, it was possible that the same person may have used different usernames on different news sites, or even that persons were creating, and posting under, multiple accounts on the same news site to bolster the perceived support of a particular viewpoint. Finally, although we attempted to be comprehensive in our search, because we only included sites that allowed comments we missed some media sources, particularly smaller local news sites without reader comments. However, these types of sites would most likely have generated a small volume of comments based on the readership size. This biases the results towards articles on larger media sources that allow commenting. We used online newspaper articles’ comments as our sole source of public perception data. Future studies could consider other social media such as Facebook, Twitter, and blogs to confirm findings. Finally, our study was conducted in 2013, reflecting opinions prior to the policy being upheld in court, and therefore may not reflect current opinions. We only searched English-language articles, and given BC’s large East Asian and South Asian populations, this is a limitation on the generalizability of these results. This topic has not been addressed by the French-language media in Quebec and BC does not have a large Francophone population.

## Conclusion

Our study identified a variety of themes in the public perception of the 2012 BC HCW condition of service influenza vaccine policy. The majority of comments did not support the policy, and a plurality of comments contained negative sentiment towards influenza vaccines. There was alarm over the perceived lack of freedom of choice in the policy, and concern about vaccine effectiveness and the perceived lack of transparency in the relationship between government and industry. However, it was also recognized that patient safety is an important part of the conversation. These findings indicated key areas of public communication that need to be addressed by health or government officials as they implement similar HCW vaccination strategies. They also suggest the potential need for efforts at counter-messaging on online comment boards or on social media. Such communication efforts by health or government officials should emphasize the following: 1) the choice given in such policies; 2) the science behind the decision, such as the effectiveness and safety of influenza vaccines, particularly in the healthcare setting; 3) benefits of the policy such as improved patient and worker safety; and 4) the limits on public health interactions with the pharmaceutical industry and how this is enforced. A future study looking at comments on articles about the BC policy post-implementation may reveal if attitudes among commenters have shifted.

## Supporting Information

S1 Fig Number of times a source is cited (if n>1), divided by sentiment. Cochrane Collaboration reports and a Center for Infectious Disease Research and Policy report were the two most cited sources of information in the comments.
(PDF)

S1 Table Eligible Canadian news articles. (PDF)

S2 Table Source Comments. Raw data of the 1163 comments and 648 users with preliminary stats. (XLSX)
