# Supplementary material for: Examining Perceptions about Mandatory Influenza Vaccination of Healthcare Workers through Online Comments on News Stories
Source: PLoS One. 2015 Jun 18;10(6):e0129993. doi: 10.1371/journal.pone.0129993 (PMC4473076; doi:10.1371/journal.pone.0129993)
Supplement: S1 Fig — Cochrane Collaboration reports and a Center for Infectious Disease Research and Policy report were the two most cited sources of information in the comments. (PDF) [file pone.0129993.s001.pdf]

S1 Figure: Number of times a source is cited (if n>1), divided by sentiment

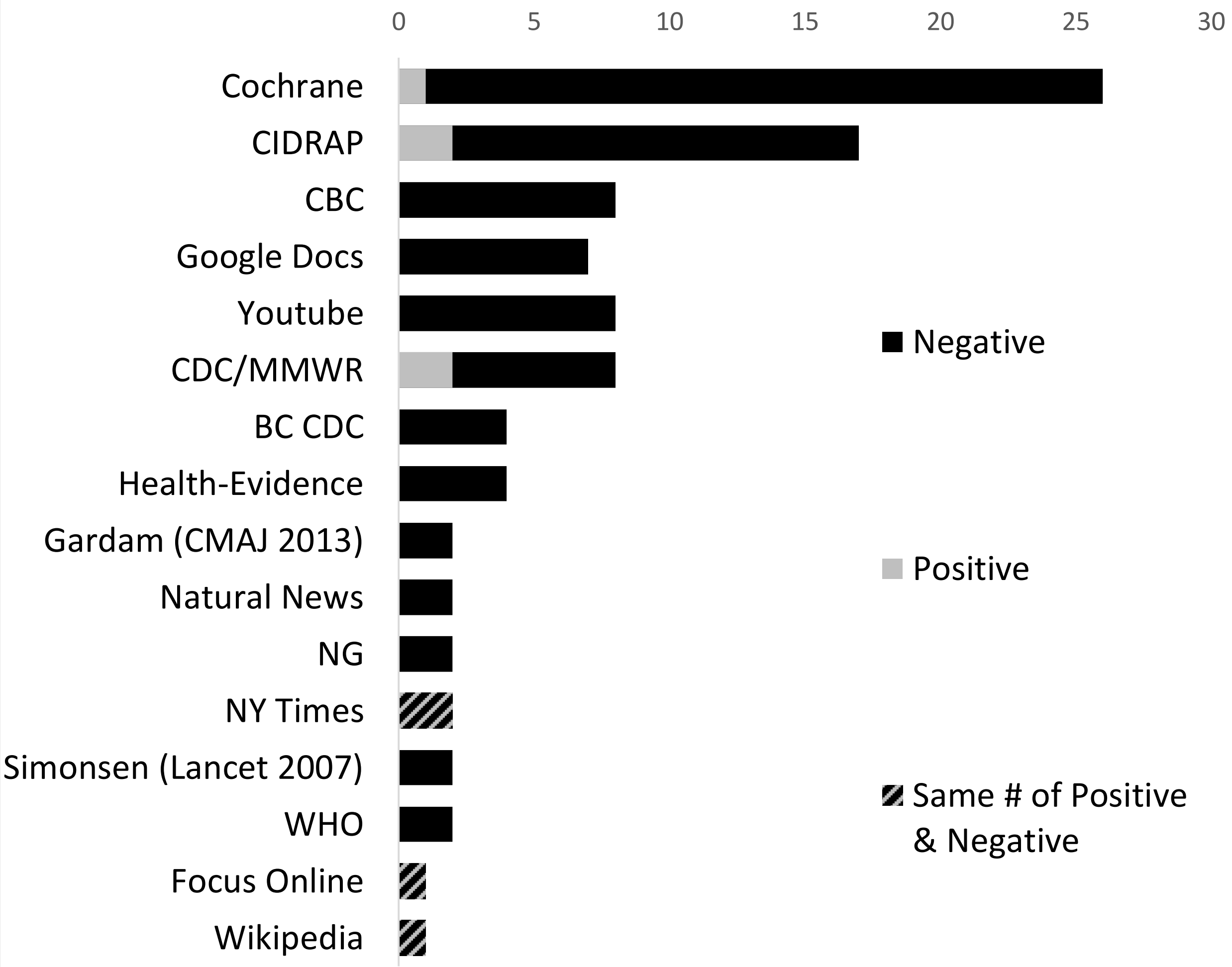

CIDRAP = Center for Infectious Disease Research and Policy
CBC = Canadian Broadcasting Corporation
CDC/MMWR = Centers for Disease Control and Prevention: Morbidity and Mortality Weekly Report
BC CDC = British Columbia Centre for Disease Control
WHO = World Health Organization

Google Docs – "Masking the Problem"
https://sites.google.com/site/vaccinesandmasks/
CIDRAP – The Compelling Need for Game- Changing Influenza Vaccines
http://www.cidrap.umn.edu/sites/default/files/public/downloads/ccivi_report.pdf
