# Supplementary material for: Examining Perceptions about Mandatory Influenza Vaccination of Healthcare Workers through Online Comments on News Stories
Source: PLoS One. 2015 Jun 18;10(6):e0129993. doi: 10.1371/journal.pone.0129993 (PMC4473076; doi:10.1371/journal.pone.0129993)
Supplement: S1 Table — (PDF) [file pone.0129993.s002.pdf]

S1 Table: Eligible Canadian news articles

Agency	Article Title	WebCite	# of comments	Date
Canadian Broadcasting Corporation (CBC)	**Compulsory flu shots for health workers called 'premature'**	http://www.webcitation.org/6HWGqndVg	86	March 25, 2013
CBC	**BC suspends health workers' mandatory flu shots**	http://www.webcitation.org/6HWH3iEQq	5	December 4, 2012
CBC	**Flu shots for hospital workers enforced in U.S.**	http://www.webcitation.org/6HWIAiBGA	10	January 15, 2013
CBC	**Mandate flu vaccines for health-care workers, journal says**	http://www.webcitation.org/6HWIHp8Z2	175	October 29, 2012
CBC	**Hospital flu shot policy 'a little bit draconian'**	http://www.webcitation.org/6HWIJSaCK	14	January 30, 2013
The Globe & Mail (GM)	**Doctor stirs debate over mandatory flu shots**	http://archive.is/nivwk	22	March 25, 2013
GM	**Health workers should make flu shot a point of pride**	http://archive.is/hUXb8	14	January 30, 2013
GM	**Health-care workers cheer softened stand on flu shots**	http://archive.is/C4Uum	19	December 3, 2012
GM	**Flu shot should be mandatory, journal says**	http://archive.is/7VxXb	37	October 29, 2012
GM	**New flu season policy targets B.C. health workers**	http://archive.is/5FYE4	10	August 23, 2012
The National Post (NP)	**Nurse's aide sent home for refusing flu shot the latest healthcare worker suspended in push to make immunization mandatory**	http://www.webcitation.org/6HWItcE2r	233	January 24, 2013
NP	**Union cites possible 'philosophical or religious objections' as B.C. nurses balk at forced flu vaccinations**	http://www.webcitation.org/6HWIwTtDP	119	October 23, 2012
NP	**Hold off on mandatory flu shots for health workers until vaccine is more effective: Toronto specialist**	http://www.webcitation.org/6HWIyzaZL	13	March 25, 2013
CTV Television Network (CTV)	**Debate over mandatory flu shots for health-care workers premature: expert**	http://www.webcitation.org/6HWJ8XlpF	9	March 25, 2013
CTV	**U.S. hospitals grapple with crackdown on workers refusing flu shots**	http://www.webcitation.org/6HWJA8TPo	15	January 12, 2013
CTV	**Should the flu shot be mandatory for health care workers?**	http://www.webcitation.org/6HWJBVG7A	67	January 4, 2013
CTV	**B.C. Nurses' Union continues fight over mandatory flu shots**	http://www.webcitation.org/6HWJDCHmm	17	November 15, 2012

CTV	**Op-ed adds ammo to fight over mandatory flu shots**	http://www.webcitation.org/6HWJFToNJ	12	November 14, 2012
CTV	**Flu shots should be mandatory for hospital workers: CMAJ**	http://www.webcitation.org/6HWJHyIax	68	October 29, 2012
CTV	**Health care workers face firing over flu shots: union**	http://www.webcitation.org/6HWJOuPpm	26	October 24, 2012
CTV	**Mandatory flu shots concern B.C. health-care workers**	http://www.webcitation.org/6HWJQLaTX	19	August 24, 2012
Coast Reporter	**Mandatory flu shot concerns nurses**	http://www.webcitation.org/6HWJRer3K	2	August 31, 2012
Comox Valley Record	**Flu edict for health-care workers will not be enforced**	http://www.webcitation.org/6HWJTuWjQ	1	December 6, 2012
Cowichan News Leader	**It will be needles or masks for front-line workers in local health care operations**	http://www.webcitation.org/6HWJWZqEk	1	September 1, 2012
The Kamloops Daily News	**Flu shot requirement relaxed**	http://www.webcitation.org/6HWJYPiOE	18	December 5, 2012
The Kamloops Daily News	**Mandatory shots asking for trouble**	http://www.webcitation.org/6HWJZOm9y	20	November 19, 2012
The Kamloops Daily News	**Health-care workers face stricter flu policies**	http://www.webcitation.org/6HWJaa1tq	3	August 23, 2012
The Kamloops Daily News	**Unions, health officials to meet over new flu policy**	http://www.webcitation.org/6HWJbMKAq	23	May 17, 2013
Kamloops This Week	**Too many health-care workers immunized against knowledge**	http://www.webcitation.org/6HWJcXwGu	4	August 27, 2012
Surrey Leader	**Health union fighting needle-or-mask flu edict**	http://www.webcitation.org/6HWJdZbvM	4	October 25, 2012
The Tyee	**BC backing down on flu shot requirement, says BCNU**	http://www.webcitation.org/6HWJeTHmq	21	November 30, 2012
The Tyee	**Why Your Nurse Didn't Have to Take a Flu Shot**	http://www.webcitation.org/6HWJfCr5a	33	February 23, 2012
The Tyee	**BC nurses concerned about choice of flut shots or mask**	http://www.webcitation.org/6HWJgFi3J	23	August 24, 2012
The Vancouver Sun	**Editorial: healthcare workers should get the flu shot**	http://www.webcitation.org/6HWJhAv4h	3	April 17, 2013
The Vancouver Sun	**Editorial: Don’t let dispute fool you — get a flu shot**	http://www.webcitation.org/6HWJjyj3W	5	November 16, 2012
The Vancouver Sun	**B.C. to require health-care workers to get flu shot or wear masks**	http://www.webcitation.org/6HWJksAcS	12	August 24, 2012
